# Hyperuricemia and Gout are Associated with the Risk of Atrial Fibrillation: An Updated Meta-Analysis

**DOI:** 10.31083/j.rcm2305178

**Published:** 2022-05-16

**Authors:** Yingjian Deng, Qiang Li, Faguang Zhou, Binni Cai, Jincun Guo, Guiyang Li, Linlin Li, Xin Su, Jianghai Liu, Dong Chang

**Affiliations:** ^1^Department of Cardiology, Xiamen Cardiovascular Hospital of Xiamen University, School of Medicine, Xiamen University, 361000 Xiamen, Fujian, China

**Keywords:** hyperuricemia, gout, atrial fibrillation, systematic review, meta-analysis

## Abstract

**Background:**

Although it has been suggested that hyperuricemia and gout are predictive of the future risk of atrial fibrillation, there is still a lack of epidemiological evidence.

**Objective:**

Through an updated systematic review and meta-analysis, we aimed to assess the association between hyperuricemia/gout and atrial fibrillation.

**Methods:**

We performed a systematic search of EMBASE, PubMed, and Web of Science from their establishment to September 2021 for all relevant studies of hyperuricemia or gout and atrial fibrillation. Meta-analysis was conducted using the random-effects method to calculate the overall relative risk (RR) and 95% confidence intervals (CI), and subgroup analyses were performed on data subsets by geographic location and study design.

**Result:**

A total of 12 studies were included in this study. The results from 8 studies showed that hyperuricemia was associated with an increased incidence of atrial fibrillation (RR: 1.83, 95% CI: 1.35–2.47), but significant association was only observed in studies in China (RR: 1.88, 95% CI: 1.31–2.71) and cross-sectional studies (RR: 2.35, 95% CI: 1.97–2.81) rather than studies in Japan (RR: 1.74, 95% CI: 0.71–4.23) and cohort studies (RR: 1.20, 95% CI: 0.99–1.46). The results from 4 studies showed that gout was also associated with an increased risk of AF (RR: 1.33, 95% CI: 1.04–1.71).

**Conclusions:**

Hyperuricemia and gout are associated with an increased incidence of atrial fibrillation.

## 1. Introduction

Atrial fibrillation (AF) is the most common cardiac arrhythmia, with increasing prevalence and incidence globally [1, 2]. In addition to high incidence rates of stroke and disabilities, AF is also associated with exacerbation of heart failure and significant mortality, imposing a substantial socioeconomic burden on the whole society [3, 4]. The causes of AF are multi-factorial and complex, and the pathogenesis of AF still remains unclear. Some risk factors of AF have been well established, such as aging, male sex, hypertension, obesity, sleep apnea, valve diseases, left ventricular dysfunction, and alcohol consumption [5, 6]. The understanding as well as reduction of risk factors for atrial fibrillation is critical for public health and clinical practice.

Uric acid is a final product of purine metabolism and circulates in blood as urate. Hyperuricemia is caused by the imbalanced formation and excretion of uric acid, which has been recognized as the underlying cause of gout. Both hyperuricemia and gout are regarded as inflammation factors [7]. The prevalence of hyperuricemia has been increasing rapidly over years [8], and its relationship with cardiovascular diseases has been widely concerned. A growing body of evidence suggests that hyperuricemia and gout are independent cardiovascular risk factors [9, 10, 11]. Recently, the relationship of hyperuricemia and gout with AF has also been revealed. Previous studies suggested that hyperuricemia and gout were associated with AF [12, 13, 14]. However, some prospective studies showed conflicting results, such as positive associations only among females [12]; the study of Wang *et al*. [15] found that uric acid was not associated with the recurrence of AF.

A previous meta-analysis of 6 cohort studies suggested that hyperuricemia was associated with a significantly increased risk of atrial fibrillation [16]. In a meta-analysis by Stella *et al*. [17], the association between uric acid and AF was also significant, indicating that the serum uric acid level in patients with AF is significantly higher, as compared to those without atrial fibrillation. With the emergence of new studies in recent years, we may need an updated systematic review and meta-analysis to further verify this relationship. Therefore, we conducted this meta-analysis with the use of the latest studies to investigate whether hyperuricemia and gout are associated with an increased risk of atrial fibrillation.

## 2. Methods

### 2.1 Search Strategy

We performed a systematic search of EMBASE, PubMed, and Web of Science from their establishment to September 2021, based on the following MESH terms and keywords: “AF” OR “Atrial fibrillation” and “Hyperuricemia” OR “Uric acid” OR “Urate” OR “Gout” (see **Supplementary Table 1**). We also manually searched citations of the identified articles and reports for potentially eligible studies. There was no restriction on languages or geographic regions.

### 2.2 Inclusion and Exclusion Criteria

**Fig. 1.**
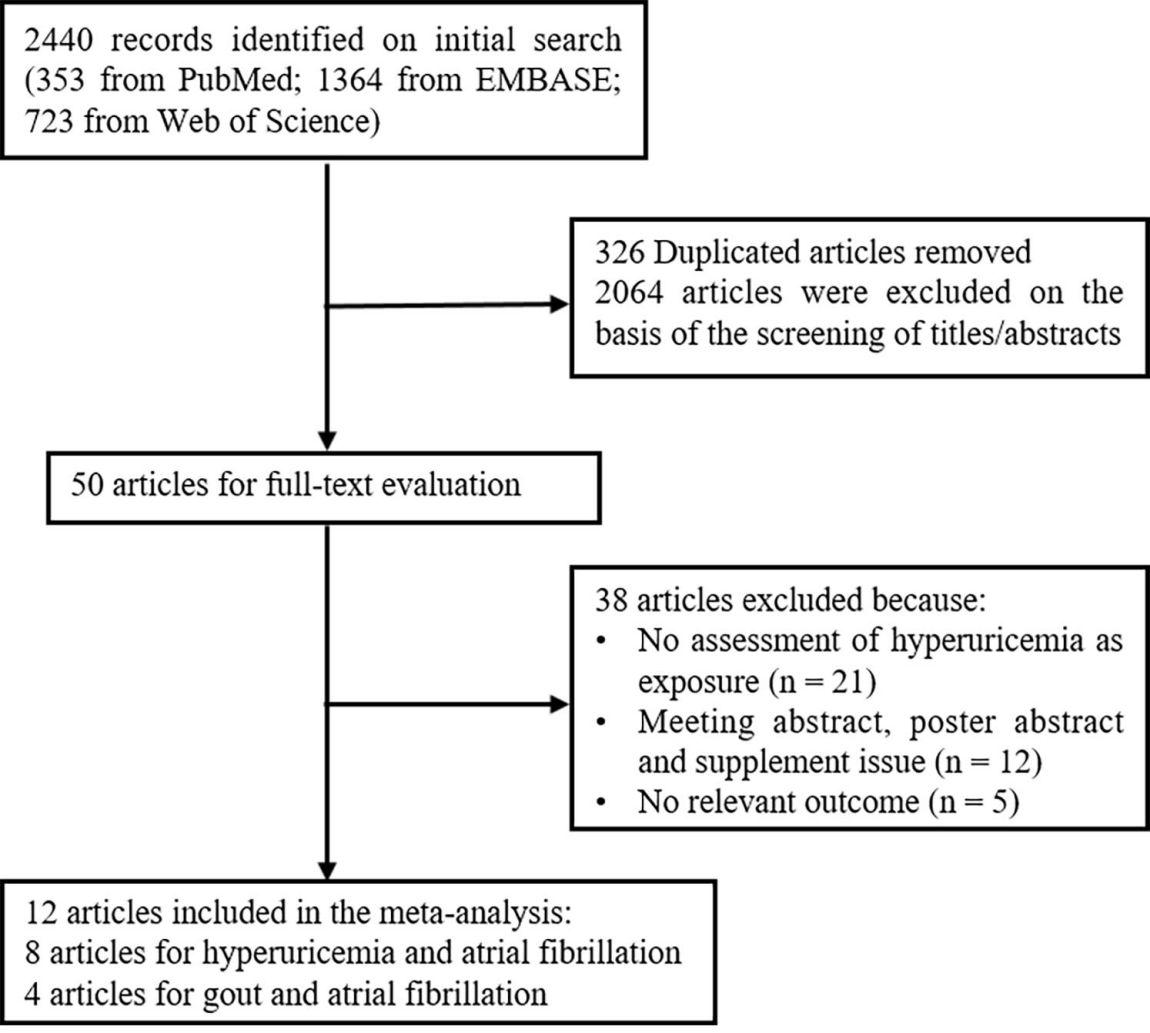
**Flow diagram of study selection process**.

The articles were assessed by two independent researchers (G.Y.L and J.H.L), in accordance with the MOOSE (meta-analysis of observational studies in epidemiology) guidelines [18]. Twelve studies were selected based on the following inclusion criteria: (1) studies on the association between hyperuricemia or gout and the risk of AF; (2) observational studies; and (3) studies with enough information to determine relative risk (RR), hazard ratio, or odds ratio with a 95% confidence interval (CI). Letters, editorials, ecological studies, comments, reviews, meta-analyses, and RCTs were excluded. Unpublished papers were also excluded in our meta-analysis (Fig. 1).

### 2.3 Quality Assessment

The methodological quality of the studies was assessed independently by two authors (Q.L and J.C.G). The Newcastle-Ottawa scale for cohort studies was used for the quality assessment of cohort studies (**Supplementary Table 3**). The case-control and cross-sectional studies were evaluated using a modified version of the Newcastle-Ottawa scale [19] (**Supplementary Table 4**). The quality of the studies was rated on a scale from 0 to 9 (0 = high risk of bias; 10 = low risk of bias). Data were extracted by one author (Q.L) and checked by another author (J.C.G) to ensure accuracy. Any disagreements were resolved by discussion and agreement among all reviewers.

### 2.4 Statistical Analysis

The adjusted RR values reported in the selected studies were used to calculate the pooled RR. The pooled ratios and their 95% confidence intervals were reported for outcome analyses. Homogeneity was tested using the Cochran’s Q-statistic and $I^{2}$ tests, where *p *< 0.05 and $I^{2}$ > 50% suggested significant heterogeneity [20, 21]. Descriptive statistics included percentages and means. Furthermore, data were subset by geographic location (Japan vs China) and study design (cohort vs cross-sectional) for subgroup analyses. The results were displayed using forest plots with effect size and 95% confidence intervals. Potential publication bias was assessed by visual inspection of the Begg funnel plot, with more symmetrical distribution of dots in the funnel plot suggesting lower degrees of publication bias. To evaluate the influence of each study on the pooled estimates, we conducted sensitivity analyses by excluding each study one by one. All the analyses and meta-analyses were performed using the Stata software, version 13 (StataCorp, College Station, TX, USA). Some images were processed using Review Manager version 5.1 (Cochrane Collaboration, Oxford, UK).

## 3. Results

### 3.1 Description of the Studies 

A total of 2440 studies were screened out from the electronic databases (353 from PubMed, 1364 from EMBASE, and 723 from Web of Science). We excluded 326 duplicate articles based on their titles and abstracts. And then, 2064 studies were excluded after screening the title and abstracts and 50 articles remained for full-text evaluation. Among them, 38 articles were further excluded due to various reasons. Finally, a total of 12 studies (8 on the association of hyperuricemia and AF and 4 on the association of gout and AF) were included in the present meta-analysis, with 2 studies performed in the USA, 2 in the UK, 2 in Japan, and 6 in China. All subjects were adults. The cut-off threshold of the level of uric acid for the determination of hyperuricemia ranged from 410–420 μmol/L (6.9–7.0 mg/dL) in males and 340–360 μmol/L (5.7–6.0 mg/dL) in females. Results from the 12 studies were adjusted for the traditional AF risk factors such as age, sex, and BMI. The majority (58.3%) of the 12 selected studies were of high quality (**Supplementary Tables 3**,**4**). The process of study selection is illustrated in Fig. 1, and the characteristics of included studies in the meta-analysis are shown in **Supplementary Table 2**.

### 3.2 Hyperuricemia and AF Risk

**Fig. 2.**
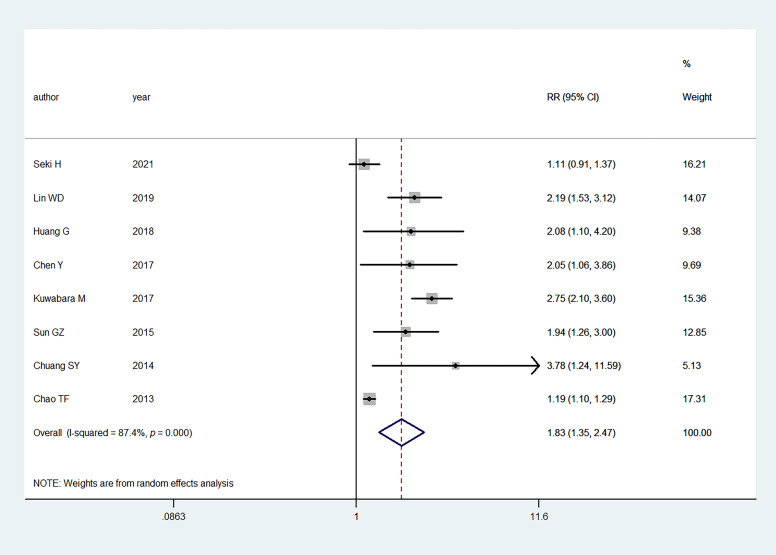
**Primary outcome of meta-analysis: association between hyperuricemia and AF**. RR, relative risk; CI, confidence interval.

Eight studies evaluated the association between hyperuricemia and the risk of AF. In the pooled analysis, hyperuricemia was associated with an increased AF risk (RR: 1.83, 95% CI: 1.35–2.47); the heterogeneity of the studies was significant ($I^{2}$ = 87.4%, *p *< 0.001) (Fig. 2).

### 3.3 Gout and AF Risk

**Fig. 3.**
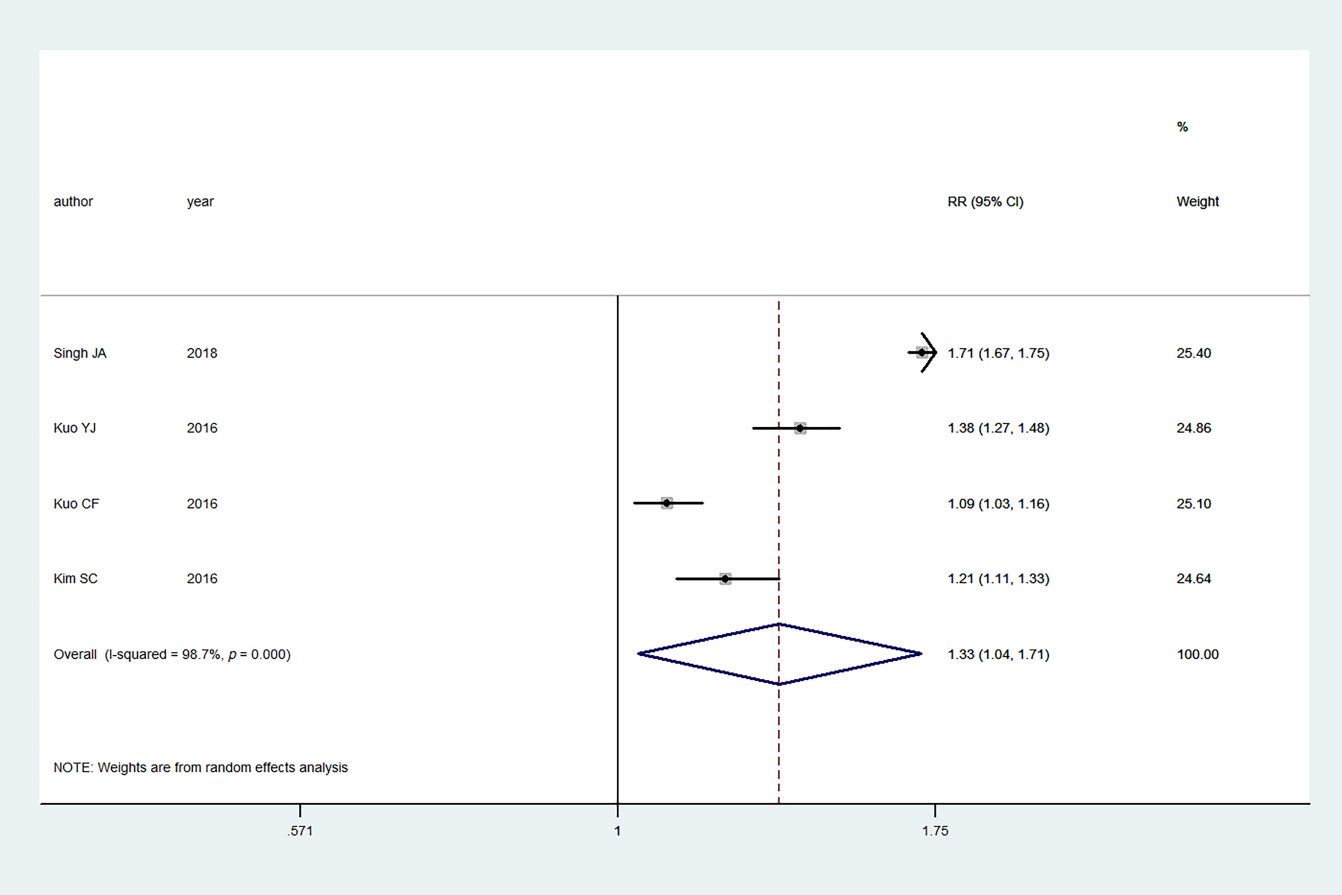
**Primary outcome of meta-analysis: association between gout and AF**. RR, relative risk; CI, confidence interval.

Four studies investigated the association between gout and the risk of AF. Overall, gout was also associated with an increased risk of AF (summary RR: 1.33, 95% CI: 1.04–1.71); there was also significant heterogeneity among the studies ($I^{2}$ = 98.7%, *p *< 0.001) (Fig. 3).

### 3.4 Subgroup Analysis

**Fig. 4.**
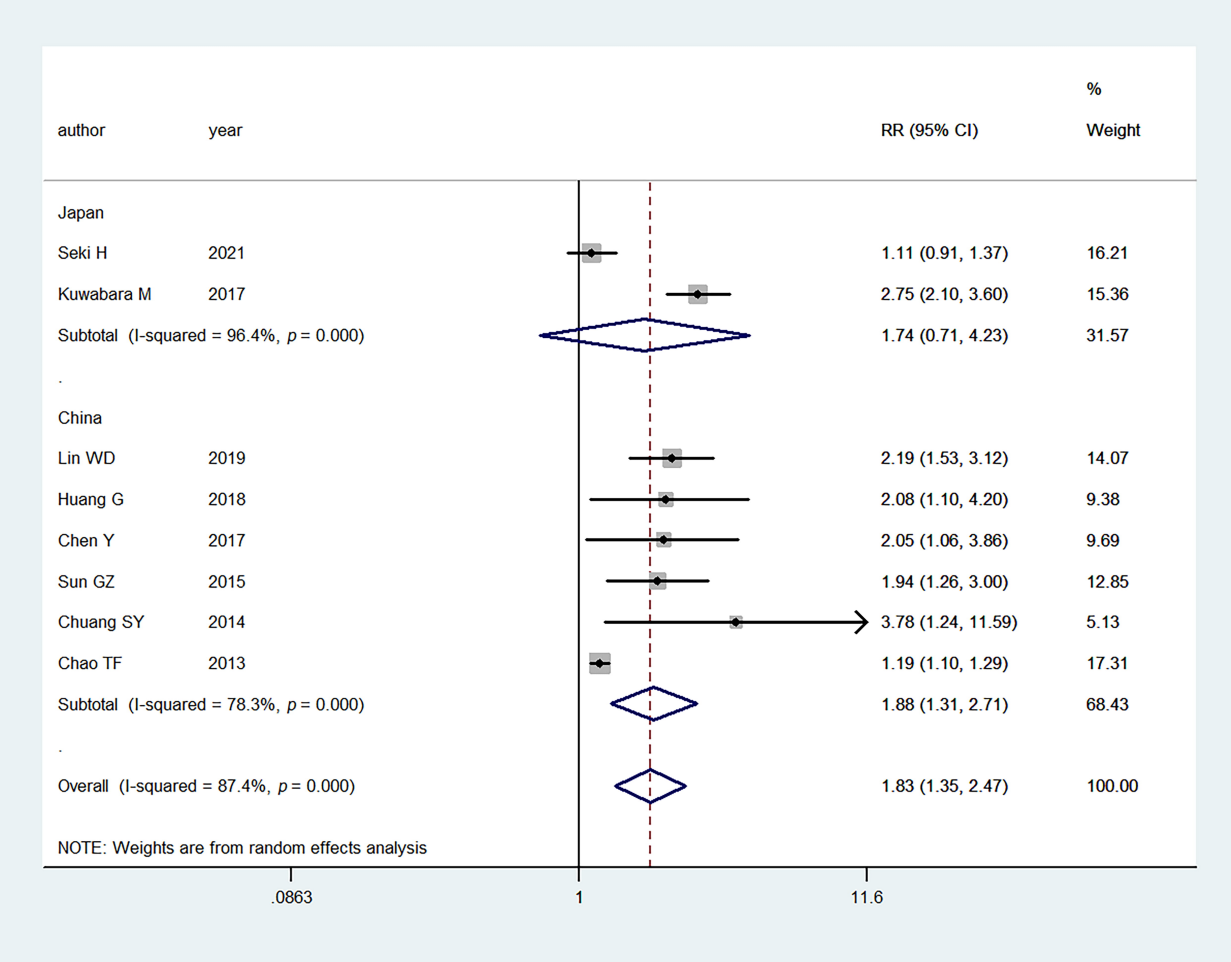
**Meta-analysis of the association between hyperuricemia and AF based on geographic location**. RR, relative risk; CI, confidence interval.

**Fig. 5.**
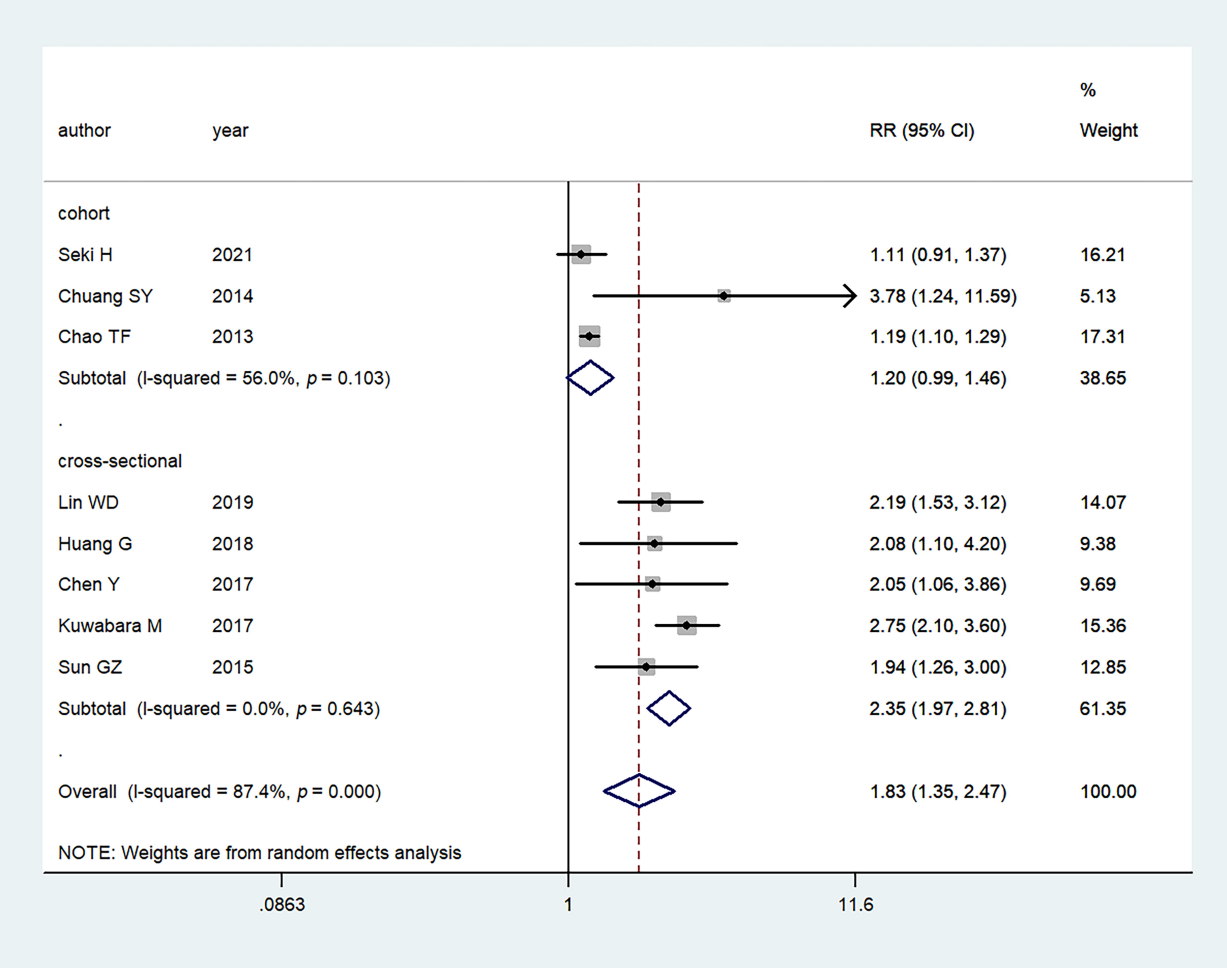
**Meta-analysis of the association between hyperuricemia and AF based on study design**. RR, relative risk; CI, confidence interval.

We performed subgroup analysis based on the geographic location (Japan vs China) and study design (cohort vs cross-sectional). When stratified by geographic location, the studies showed that hyperuricemia was not significantly associated with the AF risk in the studies in Japan (RR: 1.74, 95% CI: 0.71–4.23) (Fig. 4). However, studies in China presented a significant association between hyperuricemia and the risk of AF (RR: 1.88, 95% CI: 1.31–2.71) (Fig. 4). With regard to the study design, the association was not significant in the cohort studies (RR: 1.20, 95% CI: 0.99–1.46), while it was significant in the cross-sectional studies (RR: 2.35, 95% CI: 1.97–2.81) (Fig. 5).

### 3.5 Publication Bias and Sensitivity Analysis

**Fig. 6.**
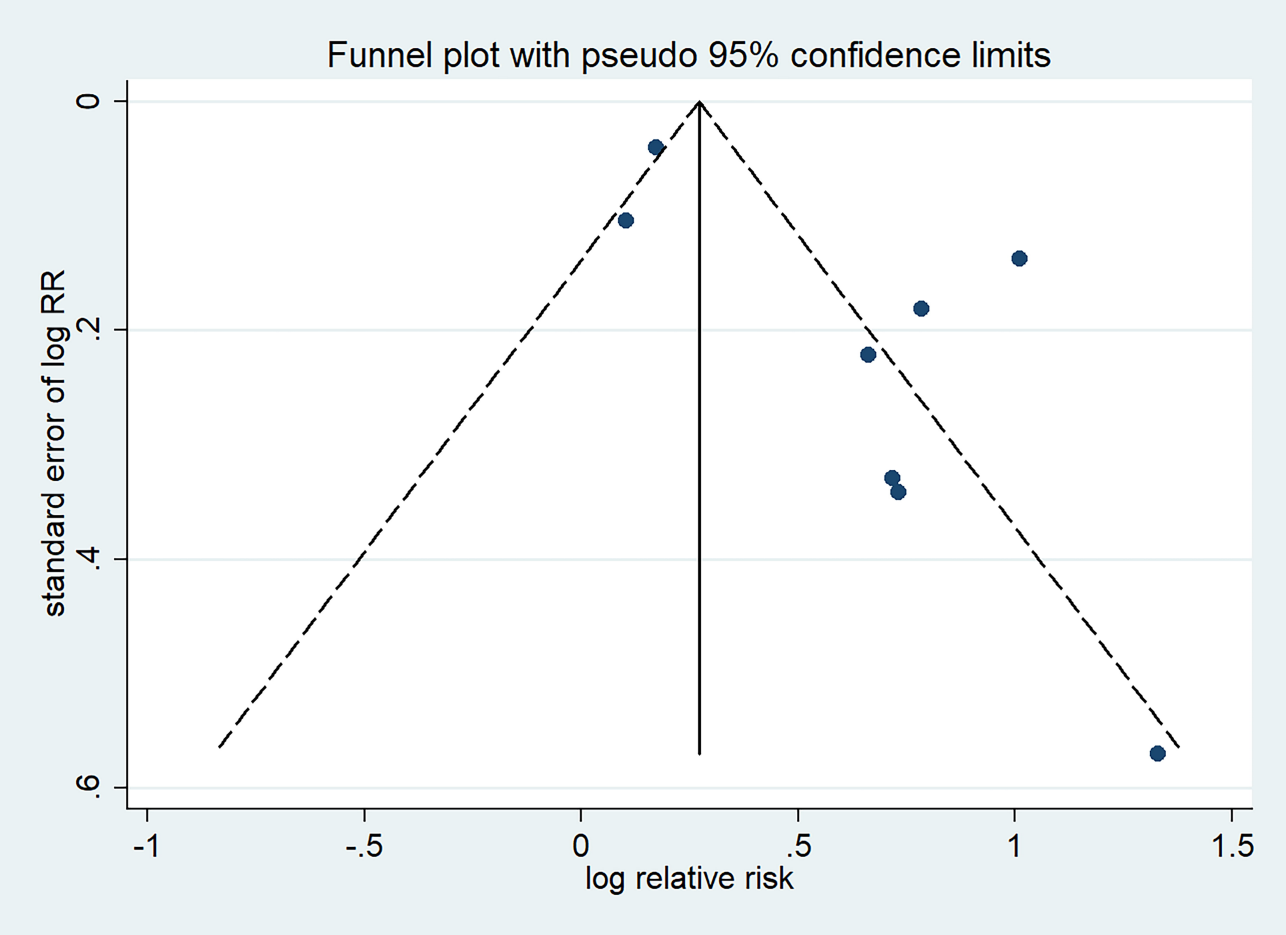
**Funnel plot of publication bias for hyperuricemia with risk of AF (Egger *p* = 0.038)**. RR, relative risk.

**Fig. 7.**
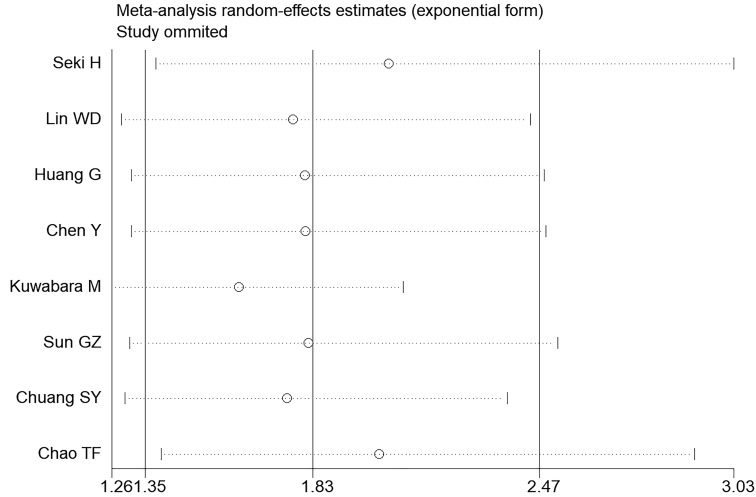
**Sensitivity analysis of the summary odds ratio coefficients for the association between hyperuricemia and AF**.

Potential publication bias was graphically evaluated via visual inspection of the funnel plot, which showed that the studies were distributed fairly symmetrically and predominantly within pseudo 95% confidence limits (Fig. 6). The sensitivity analysis revealed that the pooled RR was not substantially altered by any individual study (Fig. 7).

## 4. Discussion

This systematic review and meta-analysis further confirmed that hyperuricemia and gout were significantly associated with AF, and were independent risk factors for AF in addition to the traditional factors. To our knowledge, this is the first study including a subgroup analysis based on geographic locations; the comparison showed that the association was significant for studies in China but insignificant for studies in Japan, suggesting that the relationship might be related to geographical factors.

The major finding of this systematic review and meta-analysis of the latest literature was that both hyperuricemia and gout are associated with AF, yet with differences across geographic regions, which further clarified the association suggested in previous studies. For example, a cohort study in Taiwan by Chao *et al*. [14] suggested that the incidence of AF was higher in patients with hyperuricemia than those without (2.1% vs 1.7%; *p *< 0.001). Chuang *et al*. [22] also found that hyperuricemia was associated with AF in a healthy population (age-adjusted hazard ratio [HR] = 2.65). Moreover, a study based on UK data by Kuo *et al*. [13] reported that the cumulative probability of AF was 1.08 at 1 year, 2.03 at 2 years, and 4.77 at 5 years in patients with gout, while the figures were 0.43, 1.08, 2.95 in controls, respectively. A cohort study from the US evaluating the incidence of AF in patients with gouts and osteoarthritis also revealed a modest increase in the incidence of AF in patients with gout after adjusting for other potential risk factors, as compared with those without gout [23]. In a meta-analysis including 7 cohort studies (146,792 participants, in total), Xu *et al*. [24] reported that hyperuricemia was independently and significantly associated with an increased risk of AF (RR = 1.80, 95% CI 1.37–2.38, *p *< 0.00; $I^{2}$ = 81.6%). Similarly, in a meta-analysis of the relationship between gout and AF, Leung *et al*. [25] found that gout was significantly associated with an increased risk of AF (HR = 1.31, 95% CI: 1.00–1.70; *p* = 0.05; $I^{2}$ = 99%). A recent Mendelian randomization analysis in UK Biobank showed that individuals with hyperuricemia or gout are at an increased risk of cardiovascular diseases such as arrhythmia, which provides strong evidence to support the clinical data [26]. Our updated meta-analysis was consistent with previous studies and further confirmed this correlation. In recent years, studies have shown that the prevalence of hyperuricemia and gout varies across countries because of the differences in the genetic, cultural, socioeconomic, and behavioral backgrounds [27, 28]. A recent study has revealed that Chinese and Japanese populations have allele frequencies associated with a higher risk of hyperuricemia and gout, as compared with Europeans and populations in the southwest areas of the United States [28]. Interestingly, our subgroup analysis showed different results between studies conducted in China and Japan, suggesting the need for further exploration of the underlying mechanisms. Notably, the negative result of the analysis of cohort studies might be resulted from the study by Seki *et al*. [29], which included young adults (aged 20–40 years) only, as the prevalence of AF increases with age. This result should be interpreted with caution, and more large-scale clinical trials are still needed for verification.

The mechanism that hyperuricemia and gout are associated with AF is not completely understood. High levels of uric acid are recognized as an inflammatory factor in clinical practice [30]. A growing body of evidence suggests that inflammation is involved in both the initiation and maintenance of AF [31]. Previous studies suggested that hyperuricemia was associated with cardiac electrical and structural remodeling through a variety of mechanisms, such as inflammation, oxidative stress, fibrosis, apoptosis, and its associated immune responses [32, 33]. Maharani *et al*. [34] found that uric acid led to an increase in the expression of Kv1.5 protein, which plays an important role in the electrophysiology of atrial myocytes by modulating action potential repolarization and shortening the action potential duration. A high level of uric acid level has been shown to be associated with a high risk of metabolic syndrome, hypertension, and cardiovascular diseases, which are associated with the development and maintenance of AF [33, 35, 36, 37]. It was also found that the level of uric acid was associated with systolic dysfunction of the left atrial appendage [38]. There are also studies showing that hyperuricemia is associated with a high risk of AF recurrence after catheter ablation [39]. The mechanism of gout and AF, however, is still unclear. A previous study showed that the absolute risk of AF was approximately 60% higher in patients with gout than in the age- and sex-matched controls [13]. Hyperuricemia may also be the potential mechanism underlying the association between gout and AF. In addition, it has been demonstrated that gout is associated with ischemic heart disease and heart failure, both of which are well-established risk factors for AF [13, 40, 41].

Several limitations in our meta-analysis should be noted. Firstly, most of these studies were conducted in East Asia (China and Japan), which might not be representative of the general population across the world. Secondly, the number of studies and the size of samples included in this research were limited, and more studies are needed to further verify the association between hyperuricemia/gout and AF. In addition, although we conducted subgroup analysis based on geographic locations, there is still a lack of evidence for possible greater regional variations.

In recent years, there has been an increasing interest in the risk factors, pathogenetic mechanism, and prognosis of AF. However, epidemiological evidence for hyperuricemia and gout as independent risk factors for AF remains limited. More preclinical and clinical studies are still needed for further investigation in this field.

## 5. Conclusions

This meta-analysis showed that hyperuricemia and gout were associated with an increased incidence of atrial fibrillation. However, with the limitations in this study and the unknown mechanism, further studies are still needed to verify the results.

## Supplementary Material

Supplementary material associated with this article can be found, in the online version, at https://doi.org/10.31083/j.rcm2305178.
