# Supplementary material for: Hyperuricemia and Gout are Associated with the Risk of Atrial Fibrillation: An Updated Meta-Analysis
Source: Rev Cardiovasc Med. 2022 May 16;23(5):178. doi: 10.31083/j.rcm2305178 (PMC11273668; doi:10.31083/j.rcm2305178)
Supplement: Supplementary file 1 [file 2153-8174-23-5-178-s1.zip › 2153-8174-23-5-178-s1.docx]

Supplementary Table 1. Search strategy.

| Database | Search strategy | Result |
| --- | --- | --- |
| PubMed | #1: (atrial fibrillation) OR (AF)  #2: (((Hyperuricemia) OR (Uric Acid)) OR (Urate)) OR (gout)  #3: #1 AND #2 | #1: 150,876  #2: 59,736  #3: 353 |
| Web of Science | #1: (TS = (atrial fibrillation)) OR TS=(AF)  #2: (((Hyperuricemia) OR (Uric Acid)) OR (Urate)) OR (gout)  #3: (#1) AND #2 | #1: 238,119  #2: 115,146  #3: 723 |
| EMBASE | #1: 'atrial fibrillation' OR AF  #2: hyperuricemia OR 'uric acid' OR urate OR gout  #3: (#1) AND #2 | #1: 246,363  #2: 99,568  #3: 1364 |

Supplementary Table 2. Characteristics of studies included in the meta-analysis on the relationship between hyperuricemia/gout and the risk of atrial fibrillation.

| First author, year, country | Study design | Cases/cohort sizes or controls | Age, male ratio | Exposure | Estimates (95% CI) | Adjustments |
| --- | --- | --- | --- | --- | --- | --- |
| Seki H, 2021, Japan | Retrospective cohort study | 684/353,613 | 20-49, 46.9% | Hyperuricemia (Serum uric acid ≥7.0 mg/dL) | 1.11 (0.91-1.37) | Age, sex, overweight/obesity, high waist circumference, hypertension, diabetes mellitus, dyslipidemia, chronic kidney disease, cigarette smoking, alcohol drinking, and physical inactivity |
| Lin WD, 2019, China | Cross-sectional study | 175/11,313 | ≥35, 35.2% | Hyperuricemia (serum uric acid level >420 μmol/L in men and >360 μmol/L in women) | 2.19 (1.53-3.12) | Age, sex, heart failure, smoking status, alcohol consumption, central obesity, elevated FPG, elevated BP, reduced HDL and raised TG level |
| Singh JA, 2018, USA | Retrospective cohort study | 161,090/1,647,812 | 75.1 ± 7.6, 42.1% | Gout (ICD-9-CM code) | 1.71 (1.67-1.75) | Age, sex, race, cardiovascular and gout medications, hypertension, hyperlipidemia and coronary artery disease (CAD) and individual components of Charlson-Romano index |
| Huang G, 2018, China | Cross-sectional study | 55/983 | 83.6 ± 3.4, NA | Hyperuricemia (serum uric acid level >416 μmol/L in men and >357 μmol/L in women) | 2.08 (1.10-4.20) | Age, sex, smoking, obesity, hypertension, DM, PP, TG, TC and eGFR |
| Chen Y, 2017, China | Cross-sectional study | 53/8884 | 42.1±13.1, 52.4% | Hyperuricemia (serum uric acid level >7.0 mg/dL in men and >5.7 mg/dL in women) | 2.05 (1.06-3.86) | Age, sex, smoking, alcohol use, diuretics use, statins use, hypertension, diabetes, MI, TIA/stroke and dyslipidemia, heart failure and gout |
| Kuwabara M, 2017, Japan | Cross-sectional study | 291/89,825 | 46.3 ± 12.0 for controls and 63.2 ± 10.9 for cases, 49% | Hyperuricemia (Serum uric acid ≥7.0 mg/dL) | 2.75 (2.10-3.60) | Age, sex, BMI, FEV_1_/FVC, pulse rate, hypertension, diabetes mellitus, dyslipidemia, smoking, drinking habits, % volume capacity, albumin, hemoglobin, total bilirubin, eGFR, sodium and potassium |
| Kim SC, 2016, USA | Cohort study | 3,412/367,904 | Mean age: 57, 81% | Gout (ICD-9-CM code) | 1.21 (1.11-1.33) | Age, sex, comorbidities, medications and healthcare utilization |
| Kuo YJ, 2016, China | Retrospective population-based cohort study | 63,264/126,528 | Mean age: 51.29, 74.4% | Gout (ICD-9-CM code) | 1.38 (1.27–1.48) | Age, diabetes mellitus, CKD, HTN, COPD, CAD, history of cardiac or pulmonary surgery, alcoholic intoxication, sleep apnea, CHF, rheumatoid arthritis, SLE, scleroderma |
| Kuo CF, 2016, UK | Cohort study | NA/90,756 | Mean age: 62.4, 72.3% | Gout (NA) | 1.09 (1.03, 1.16) | Matched for age, sex, index year and registration year, and additionally adjusted for BMI, smoking, alcohol consumption, ischemic heart disease, heart failure, heart valve disease, hyperthyroidism and other comorbidities and medications |
| Sun GZ, 2015, China | Cross-sectional study | 139/11,338 | Mean age: 53.8, 45.6% | Hyperuricemia (serum uric acid level >7.0 mg/dL in men and >5.7 mg/dL in women) | 1.94 (1.26-3.00) | Age, gender, BMI, waist circumference, systolic and diastolic blood pressure, blood glucose, total cholesterol and triglyceride levels, smoking, drinking, myocardial infarction, low left ventricular ejection fraction, left ventricular hypertrophy, and family history of AF |
| Chuang SY, 2014, China | Cohort study | 90/1485 | ≥65, 50.7% | Hyperuricemia (serum uric acid level >416 μmol/L in men and >357 μmol/L in women) | Normal BP: 3.78 (1.24-11.59)  Elevated BP: 1.04 (0.61-1.76) | Age, sex, obesity, abdominal obesity, high triglycerides, low HDL cholesterol, high glucose, smoking, C-reactive protein, Estimated GFR, LVH, hyperthyroidism and diuretics |
| Chao TF, 2013, China | Cohort study | 2,339 /122,524 | 50.6 ± 16.5, 73.7% | Hyperuricemia (NA) | 1.19 (1.10-1.29) | Age and sex |

Supplementary Table 3. Quality of included cohort studies based on the Newcastle-Ottawa scale.

| Study | Selection | | | | Comparability | Outcome | | | Total Score |
| --- | --- | --- | --- | --- | --- | --- | --- | --- | --- |
|  | Representativeness of exposed cohort  ☆ | Selection of non-exposed cohort  ☆ | Exposure ascertainment  ☆ | Demonstration that outcome of interest was not present at start of study  ☆ | Comparability of cases and controls based on design or analysis  ☆☆ | Outcome  Assessment  ☆ | Adequate follow-up  (≥10y)  ☆ | Loss to follow-up rate (≤20%)  ☆ |  |
| Seki H, 2021, Japan | ☆ | ☆ | ☆ | ☆ | ☆☆ | - | - | - | 6 |
| Singh JA, 2018, USA | ☆ | ☆ | ☆ | ☆ | ☆☆ | ☆ | - | - | 7 |
| Kim SC, 2016, USA | ☆ | ☆ | ☆ | ☆ | ☆☆ | ☆ | - | - | 7 |
| Kuo YJ, 2016, China | ☆ | ☆ | ☆ | ☆ | ☆ | ☆ | - | - | 6 |
| Kuo CF, 2016, UK | ☆ | ☆ | - | ☆ | ☆☆ | ☆ | - | - | 6 |
| Chuang SY, 2014, China | ☆ | ☆ | ☆ | ☆ | ☆☆ | ☆ | - | - | 7 |
| Chao TF, 2013, China | ☆ | ☆ | - | ☆ | ☆ | - | - | - | 4 |

Supplementary Table 4. Quality of included case-control studies and cross-sectional studies based on the Newcastle-Ottawa scale.

| Study | Selection | | | | Comparability | Outcome | | | Total Score |
| --- | --- | --- | --- | --- | --- | --- | --- | --- | --- |
|  | Adequate case definition  ☆ | Representativeness of cases  ☆ | Selection of controls  ☆ | Definition of controls  ☆ | Comparability of cases and controls based on design or analysis  ☆☆ | Exposure ascertainment  ☆ | Same method of ascertainment for cases and controls  ☆ | Non-response rate  ☆ |  |
| Lin WD, 2019, China | ☆ | - | ☆ | ☆ | ☆☆ | ☆ | ☆ | - | 7 |
| Huang G, 2018, China | ☆ | - | - | ☆ | ☆☆ | ☆ | ☆ | - | 6 |
| Chen Y, 2017, China | ☆ | - | ☆ | ☆ | ☆☆ | ☆ | ☆ | - | 7 |
| Kuwabara M, 2017, Japan | ☆ | ☆ | ☆ | ☆ | ☆☆ | ☆ | ☆ | - | 8 |
| Sun GZ, 2015, China | ☆ | - | ☆ | ☆ | ☆☆ | ☆ | ☆ | - | 7 |
